# Plant-inspired pipettes

**DOI:** 10.1098/rsif.2017.0868

**Published:** 2018-03-14

**Authors:** Keigo Nakamura, Tetsuya Hisanaga, Koichi Fujimoto, Keiji Nakajima, Hirofumi Wada

**Affiliations:** 1 Department of Physics, Ritsumeikan University, Kusatsu, Shiga 525-8577, Japan; 2 Graduate School of Biological Sciences, Nara Institute of Science and Technology, Ikoma, Nara 630-0192, Japan; 3 Department of Biological Sciences, Osaka University, Toyonaka, Osaka, Japan

**Keywords:** water-grabbing, liverwort, capillarity, free surface flow

## Abstract

The female sex organ of the liverwort (*Marchantia polymorpha*) has a characteristic parasol-like form highly suitable for collecting water droplets containing sperm for fertilization. Motivated by this observation and using three-dimensional printing techniques, we develop a parasol-like rigid object that can grab, transport and release water droplets of a maximum size of about 1 cm. By combining experiments and scaling theory, we quantify the object's fundamental wetting and fluid dynamical properties. We construct a stability phase diagram and suggest that it is largely insensitive to properties of liquids such as surface tension and viscosity. A simple scaling argument is developed to explain the phase boundary. Our study provides basic design rules of a simple pipette-like device with bubble-free capture and drop of liquids, which can be used in laboratory settings and has applications within soft robotics. Through systematic experimental investigations, we suggest the optimal design criteria of the liverwort-inspired object to achieve maximal pipetting performance. We also provide, based on our scalable model experiments, a biological implication for the mechanistic advantage of this structure in liverwort reproduction.

## 1. Introduction

Unlike animals, plants usually do not change their habitat once rooted. This fundamental constraint forces plants and fungi to develop various forms and techniques of handling [1], using [2–6] and avoiding fluids [7], according to their lifestyle and environment [8]. Some examples have expanded understanding of fluid dynamics, capillarity, wetting and elasticity [9–15], and have inspired applications in art, engineering and architecture [16,17]. For example, lotus leaves motivated the fabrication of artificial super-hydrophobic surfaces [18,19]. Cactaceae (a plant family found in desert regions) have developed specialized spines that can effectively collect water from fog for survival in extremely dry environments [20]. In these examples, nano- or microscale surface structures control wetting properties and determine the behaviour of water on the surface of the plant. Other examples mainly rely on hydrodynamic properties that emerge at larger scales, such as the closing behaviour of floating flowers during floods, which has been used to design a petal-shaped water-grabbing object [21]. This kind of passive pipetting relies on elastocapillarity, in which thin plates deform due to surface forces at the liquid interface [22]. Thus, learning from ‘plant strategies’ has proved to be a promising way to learn how to manipulate water droplets, which is the focus of this paper.

**Figure 1.**
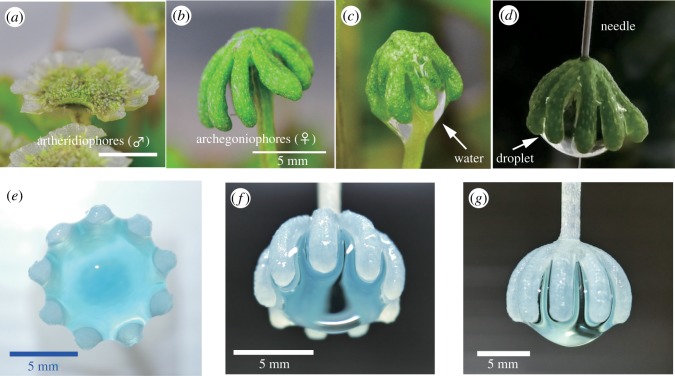
Liverwort (*Marchantia polymorpha*) (*a*) male, (*b*) female. Scale (*a*,*b*) 5 mm. (*c*) A female *M. polymorpha* grabbing water. (*d*) An ‘inverted’ model composed of a real stalk-eliminated liverwort cap with a needle capturing a droplet (when drawn out of a water bath). (*e*–*g*) Morphology of water at the maximal grabbing state in our three-dimensional printing plastic model; (*e*) view from the bottom, (*f*) and (*g*) view from the sides. (Online version in colour.)

Liverwort (*Marchantia polymorpha*) is a dioecious species with morphologically distinct male and female sex organs with parasol-like structures, known as antheridiophore and archegoniophore, respectively (figure 1*a*,*b*) [23]. If water sits in the cup-like antheridiophore, many flagellated sperms immediately disperse into the water. When the water containing sperm reaches the archegoniophores by chance, fertilization can proceed beneath the umbrella where the eggs are stored.

So far, different processes of sperm transportation have been proposed [23]^1^, including ‘splash-launch’ by raindrops [26], surfing on an air–water interface using a fat-induced surface tension gradient [27]. An entirely separate scenario from those is the transportation of sperm cells with water through the bundles of rhizoids in the stalk driven by capillarity [28,29]. The previous observation of dye-containing water drops has confirmed the occurrence of droplet splashing, but dye adsorption and spreading over the entire colony have also been observed [30], which leads to the hypothesis that the rhizoids may work as conduits for sperm cells [23].

Here we would like to draw attention to the characteristic umbrella form of the archegoniophores, which typically has 9–10 ribs that are well suited (and can be optimized) for reliable water capture (figure 1*c*,*d*). Motivated by this unique morphology, we built a scalable model using a three-dimensional printer, and conducted thorough systematic experiments wherein our geometrically adjustable device can grab, transport and release a precise amount of water whose size can exceed the capillary length. Such a large water droplet, when simply dangled, might drop due to the dominance of gravity over surface forces. The proposed method is stable, highly reproducible and reusable; the device is rigid, and its key function relies only on the interaction between its geometry and interfacial fluid forces. Hence, our study introduces a basic design of a simple pipette-like model that could be used in laboratories, independent of any elastic effects such as suction by negative pressure or elastocapillarity.

## 2. Experiments

**Figure 2.**
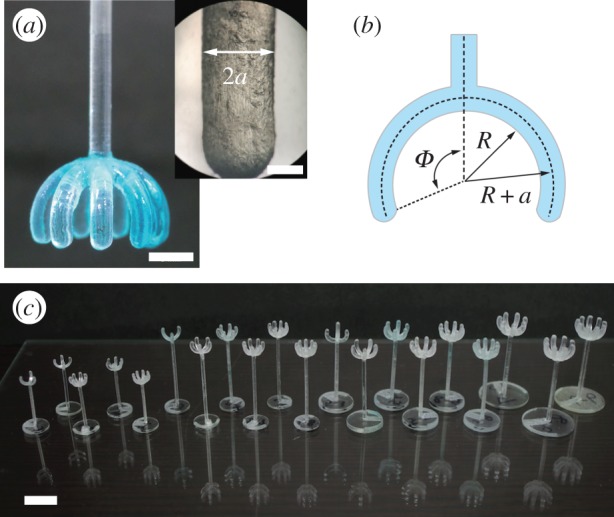
(*a*) Left pane: Photograph of the device motivated by liverwort, with *R* = 5.0 mm and *Φ* = 110°. Scale bar: 5mm. Right pane: magnified image of the surface of a rib, taken by stereomicroscope (Olympus, Japan). Scale bar: 1mm. (*b*) Definition of shape parameters *Φ*, *R* and *a*. (*c*) Photograph of a collection of our plastic models for various radii *R* and number of ribs *N*. Scale bar: 10 mm. (Online version in colour.)

To observe a simple meniscus and highlight a potential application, we considered an *inverted* configuration (figure 1*e*,*f*). A real liverwort cap with a needle replacing the stalk (figure 1*d*) can be used to grab a substantial amount of water. However, to precisely control geometric parameters, we developed an analogue model as shown in figure 2*a*. For the fabrication of such plastic models, a basic, idealized form imitating the liverwort structure was first designed using computer software (Autodesk 123D), and then three-dimensional-printed using acrylate resin (Objet 30 Pro, Stratasys, USA). The resultant envelope is a spherical surface with outer and inner surface radii of *R* + 2*a* and *R*, respectively, and a covering angle, *Φ*, ranging from 60° to 160° (figure 2*b*). Models with various inner radii, *R* (2.5 ≤ *R* < 5 mm), and rib numbers, *N* (3 ≤ *N* ≤ 9), were fabricated (figure 2*c*).

In principle, the cap radius, *R*, and the rib radius, *a*, are independent parameters. For the sake of simplicity, we varied rib radii, *a*, according to *a*/*R* = 0.2, which ensures shape similarity of devices of different sizes (figure 2*c*). The water is partially wetting to acrylate resin, and its contact angle was measured independently (table 1), which might include effects due to surface roughness (figure 2*a* inset) [31,32].

**Table 1.** Mass density (*ρ*), surface tension (*γ*), viscosity (*η*), capillary length (*κ*^−1^) and (apparent) contact angle (*θ*_*E*_), for the different types of liquids used.

liquid	*ρ* (kg m^−3^)	*γ* (mN m^−1^)	*η* (Pa s)	*κ*^−1^ (mm)	*θ*_*E*_
water	10^3^	72	10^−3^	2.7	66°−75°
ethanol	789	22	1.2 × 10^−3^	1.7	<10°
olive oil	911	32	8.4 × 10^−2^	1.9	25°−32°

To test different surface tensions and viscosities, different liquids were tested, including ultrapure and tap water, dehydrated ethanol and olive oil (Bosco, Nissin Oillio). The relevant physical parameters of these liquids are summarized in table 1 for reference. A plastic liverwort was gently immersed in a liquid bath well below the air–liquid interface, with its position precisely controlled using a stepping motor (Oriental Motor, Japan). This initial configuration ensured an upward pulling with constant velocity across the air–liquid interface. The withdrawal velocity *v* was changed in the range of 2 × 10^−3^ < *v* < 0.4 m s^−1^. The device was drawn out of the bath and pulled well above the air–liquid interface, where it stopped. During the deceleration, the device did not lose any of the captured liquid. (The dripping of a captured liquid upon stopping was only observed for olive oil.) The liquid remaining on the device at this stage was absorbed by a piece of dry paper, whose weight increase was immediately measured using an electric balance (Mettler Toledo, Switzerland). This semi-manual procedure gave a rather accurate measurement of the liquid mass. All data shown below are averaged over 5–10 independent experiments, and the error bars, which are smaller than symbols, are not visible.

As the device was pulled upwards from the liquid bath, it grabbed (or failed to grab) a certain amount of liquid depending on the size of the radius, *R*, the number of the ribs, *N*, and the velocity, *v*. The captured liquid mass is denoted below as *m*. It is worth noting that a fully closed cup 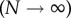 traps an air bubble inside and always fails to capture any liquid.

## 3. Results

**Figure 3.**
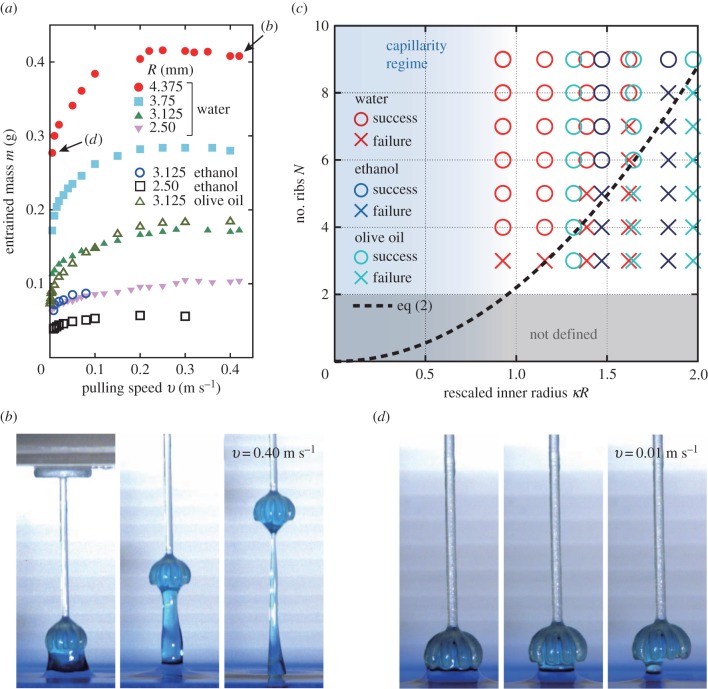
(*a*) Extracted mass, *m*, as a function of withdrawal velocity, *v*, for increasing radius, *R*, with *N* = 9 ribs in all cases. (*b*,*d*) Snapshots of typical water withdrawals for (*b*) *v* = 0.4 m s^−1^, and (*d*) *v* = 0.01 m s^−1^. The water was dyed with food colouring to enhance visualization. The corresponding mass data are indicated in (*a*) by the arrows. (*c*) Grabbing phase diagram constructed experimentally in the (*R*, *N*)-plane for different liquids (water, ethanol and olive oil) by fixing the Weber number at *We* = 6.8. Symbols indicate successful (○) and failed grabbings (×). The regime of capillarity corresponds to *κR* < 1. The dashed line is the theoretical prediction for the phase boundary given in equation (3.2). (Online version in colour.)

First, the case of *N* = 9 is discussed, as it is typically found in *M. polymorpha*. Grabbing was always successful in this case, and *m* was found to be velocity-dependent, as shown in figure 3*a*. At low velocities, *m* increased with increasing *v*, until reaching its maximum value *m*_max_, after which it remained almost constant (or slightly decreasing) in the high-velocity regime studied here. During high-velocity withdrawal, a liquid ligament developed and axially stretched as shown in figure 3*b* [33–35]. When it finally broke, its upper part was captured by the ribs (electronic supplementary material, movie S1, filmed with a high-speed camera (Ditect, Japan)). The value of *m* is maximized at this high velocity, for which the inertial force of the contained liquid, ∼*ρv*^2^*R*^2^, dominates the capillary force, ∼*γR*, where *ρ* and *γ* are the density and surface tension of the liquid, respectively. The relative significance of the two physical effects is quantified by the Weber number [36],3.1
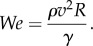
In the problems regarding the deposition of a thin liquid layer on a fibre or plate [36], the capillary number, defined as *Ca* = *ηv*/*γ*, plays an important role for velocities less than 1 m s^−1^, where *η* is the viscosity of a liquid. This is because inertial effects are relatively unimportant compared to viscous effects due to the thin geometry of a fibre or plate. In contrast with this, the typical length of our system, *R*, is 10^2^ times larger than a typical fibre size of 10–100 *µ*m, and thus *We*, as defined in equation (3.1), becomes a dominant parameter throughout our experiments.

Next, we investigate *N*-dependence. A view from below the morphology at maximum grabbing (figure 1*e*) reveals that a meniscus forms, such that the water bridges the neighbouring ribs. In mechanical equilibrium, the capillary force acting on the surface of *N* ribs, *F*_c_, supports the weight of the droplet, ∼*ρgR*^3^, where *g* is the gravitational acceleration. The former is proportional to the total length of the contact line, ∼*Nπa*, which leads to the force balance: *F*_c_ ∼ *γNa* ∼ *ρgR*^3^. Because we assume the proportionality, *a* ∝ *R*, to ensure the overall shape similarity (as explained earlier), the force balance provides the condition for successful grabbing as3.2
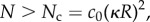
where *c*_0_ is a constant of order unity, and *κ*^−1^ = (*γ*/*ρg*)^1/2^ is the capillary length (table 1) [37]. To verify this prediction, we experimentally construct a grabbing stability phase diagram in terms of (*N*, *R*). As both *R* and *v* affect the grabbing behaviour, the diagram is constructed in figure 3*c* for a given value of *We* = 6.8 (meaning that different *v* values were applied for different *R*) for the three different liquids. Our prediction in equation (3.2) explains the phase boundary quite well, with *c*_0_ ≈ 2.2 *independent* of the type of liquid. Interestingly, this suggests that the phase boundary between successful and unsuccessful grabbing in figure 3*c* is independent of the apparent contact angle *θ*_*E*_. The largest water drop size approaches 4*κ*^−1^ ∼ 10 mm for *N* = 9, which is a few times larger than the capillary length. The mass at this maximum capture is 0.416 ± 0.001 g, or 416 mm^3^ in volume, which is a comparable value to [21]. So long as *We* > 1, diagrams for other values of *We* are confirmed to be similar to those in figure 3*c*. For *κR* < 1, the capillary force dominates gravity, and a complicated meniscus between the ribs is formed, especially for smaller *N* values, which is often difficult to interpret.

**Figure 4.**
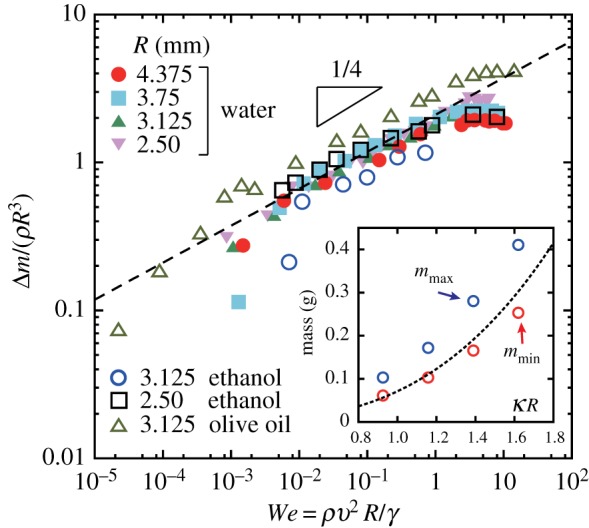
Rescaled excess mass, Δ*m*/(*ρR*^3^), as a function of Weber number, *We*, for water, ethanol and olive oil, and for various *R* values. Inset: the minimum and maximum water mass extracted from the data shown in figure 3*a*. Dotted line shows the water mass of the half-sphere of radius, *R* + *a*. (Online version in colour.)

By fixing *N* = 9, we can now discuss the low-velocity regime. Typically for *v* < (*γ*/*ρR*)^1/2^ ≈ 0.1 m s^−1^ (or *We* < 1), the surface force dominates and no significant liquid column develops during withdrawal (see figure 3; electronic supplementary material, movie 2). In slow withdrawal, the device can grab its minimum amount of liquid, *m*_min_, whenever the grab is successful. The *v*-dependence is most clearly seen by focusing on the *excess* mass, Δ*m* = *m* − *m*_min_. The minimum mass *m*_min_ was obtained by fitting the data for *We* < 1, which is shown in the inset of figure 4, together with the maximum mass *m*_max_ (which was measured directly). The dashed line represents the mass of the half-sphere of radius *R* + *a*, i.e. (2*πρ*/3)(*R* + *a*)^3^, which properly explains the *R*-dependence of *m*_min_. This confirms the absence of the air bubble trapped inside the liquid droplet in our experiments. The values of Δ*m* for the different types of liquids are shown in figure 4, from which we propose an empirical scaling relation written as3.3
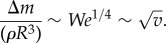
Interestingly, Δ*m* is not strongly dependent on the viscosity of the liquid. Data from the olive oil exhibit viscous corrections; however, these are quite small considering that its viscosity is almost a hundred times larger than the viscosity of water (table 1). In fact, the fast dynamics of the withdrawal of oil is remarkably different due to the formation of a high liquid column (see electronic supplementary material, movies S3 and S4) [38], which, nevertheless has a little effect on the final amount of the liquid. The physical explanation of this large independence of *m* on the liquid viscosity, as well as equation (3.3), is currently not available, and is the subject of future theoretical studies. The scaling ceases to be valid when *m* saturates at its maximum value at around *We* ∼ 1^2^.

## 4. Application

**Figure 5.**
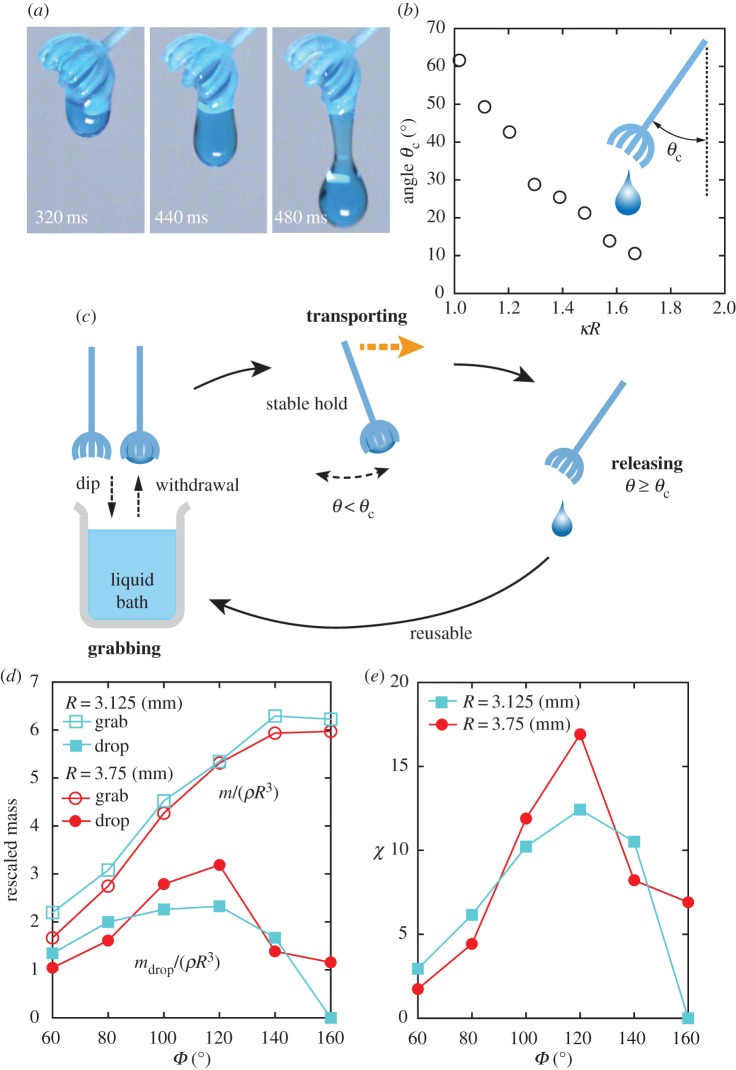
(*a*) Snapshots of a water drop falling at the critical tilting for *R* = 3.75 mm. (*b*) Experimentally determined critical tilting angle, *θ*_c_, as a function of *κR* with *N* = 9 ribs. Inset: definition of *θ*_c_. (*c*) Schematic illustration of the pipetting process using our device motivated by liverwort. (*d*) The rescaled (maximum) grabbed mass, *m*/*ρR*^3^ (open symbol) and the rescaled mass dropped at the critical tilting, *m*_drop_/*ρR*^3^ (filled symbol), plotted as a function of the shape parameter *Φ* for *R* = 3.125 mm (box) and for *R* = 3.75 mm (circle) with *N* = 9. (*e*) Pipetting efficiency *χ* = *m* × *m*_drop_/(*ρR*^3^)^2^ as a function of *Φ*. The data and the symbol legend are the same as in (*d*). All data shown here are obtained for tap water. (Online version in colour.)

An important advantage of our pipetting model is its ability to release water in a passive way. As shown in figure 5*a*, it can release water when tilted by a certain critical angle, *θ*_c_, similar to some types of honey dippers. To quantify this property, the critical angle, *θ*_c_, was measured and plotted in figure 5*b* as a function of *κR*, which demonstrates that a larger droplet flows out at a smaller angle. This process can essentially be understood by applying Tate's Law: the droplet falls when its weight overwhelms its capillary force [37,39]. However, because of the complicated geometry, an accurate description of this emptying process is challenging to develop [40]. Importantly, the experiment indicates that grabbing is sufficiently stable against small disturbances, suggesting that it allows the transportation of water to any place by hand, for example (see electronic supplementary material, movie S5) This unique property, which is absent in previous studies [10,21,22], makes our system particularly attractive as a novel pipetting tool; it provides a full sequence of processes, (i.e. grabbing, transporting and releasing in a controlled manner), as illustrated in figure 5*c*.

Pipetting performance crucially depends on the shape parameter, *Φ* (figure 2*b*). To clarify its variation with *Φ*, we investigated the grabbed mass, *m*, and the dropped mass, *m*_drop_, as a function of *Φ* for two different sizes (*R* = 3.125 and 3.75 mm) with *N* = 9. The results are shown in figure 5*d*, which reveals that a near-sphere shape (*Φ* > 140°) can grab more water, but reduces its releasing ability, as can be seen by the considerable decrease of *m*_drop_ at large *Φ*. We defined a non-dimensional quantity to measure the performance of the device as *χ* = *m* × *m*_drop_/(*ρR*^3^)^2^. The experimental *χ* is plotted as a function of *Φ* in figure 5*e*, which demonstrates that the optimal design is achieved at around *Φ* ≈ 110° − 120°. Another measure of efficiency may be the absolute amount of water that the device can transport, such as *m*_drop_, itself (figure 5*d*). Similar to the previous case, this is maximized for *Φ* ≈ 110° − 120° (and *R* = 4.375 mm). The errors in the data of *m* and *m*_drop_ are negligibly small (typically less than 10^−3^ g), which implies a stable pipetting of the exact amount of liquid. Moreover, this property seems to be independent of viscosity, which is a possible advantage over the current popular pipetting tools.

**Figure 6.**
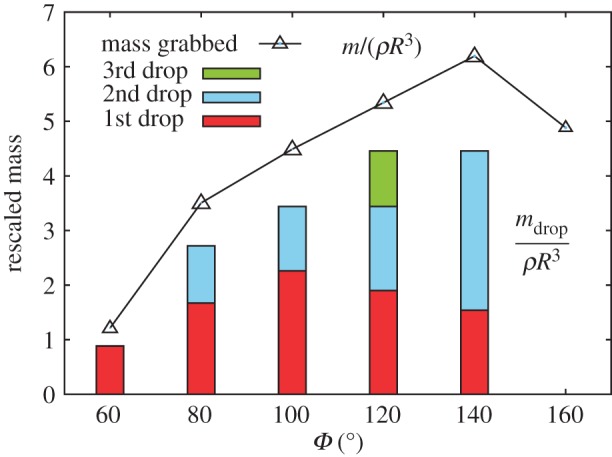
Dripping experiment with viscous olive oil. The rescaled (maximum) grabbed mass, *m*/*ρR*^3^ (open symbol) and the rescaled mass dropped at the critical tilting, *m*_drop_/*ρR*^3^ (bar), plotted as a function of the shape parameter, *Φ*, for *R* = 3.125 mm with *N* = 9. The red, blue and yellow bars represent the amount of oil at the first, second and third dripping event during a sufficiently slow increase of the tilting angle. (Online version in colour.)

However, the dropping process is somewhat more complicated for viscous fluids. In figure 6, the experimental result for olive oil is shown. Similar to that of water, the optimal design angle is around *Φ* = 120°, but for *Φ* = 80°–140°, the device exhibits liquid drops more than once (typically 2–3 times) before it is maximally tilted. This behaviour may arise from the complex pinch-off dynamics of a viscous droplet, which may require a separate, detailed study. At this stage, we stress that the amount of the viscous oil at each drop is accurate and shows little variability, again, quite like the case for water.

## 5. Discussion

Now for the biological implication of the study based on the results of the scalable model experiments. First, as shown in figure 4, the amount of water grabbed increases with *We*, and remains almost constant for *We* > 1 (which is valid at least up to *We* ∼ 10). The speed of a water drop that a female strain experiences may depend on how it is transported (e.g. splash by raindrops, free falling or wind-driven). Interestingly, it is observed that male strains grow faster, and thus may become taller than female ones, implying that free falling (triggered by raindrops or wind) could be one of the physical processes of drop transportations. If a typical vertical height difference is *h* ∼ 10 mm, drop speed is independent of its mass and is given by 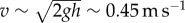, which is comparable to the highest speed we have studied here (ignoring the differences between constant-velocity and constant-acceleration motion for this rough semi-quantitative comparison). As the size of liverworts is typically *R* = 3 − 4 mm in radius [23], the associated Weber number can be estimated as *We* ∼ 8 − 10. Thus, the regime studied here may be directly relevant to the hydrodynamic conditions that wild liverworts encounter. The grabbing property induced by its shape is therefore potentially advantageous to *M. polymorpha*, as it can provide a robust and stable water delivery method, even in rain and wind, which ultimately works in favour of reproduction. Clearly, further studies are needed to explore biological aspects because the differences in the stalk position (inverted in this study) and surface structures are likely to be important.

## 6. Summary

Motivated by the shape of female *M. polymorpha*, we designed and created an umbrella-shaped centimetre-sized object by using a computer-assisted three-dimensional printing method. By combining systematic experiments with scaling theory, we demonstrated its multifunctional ability to manipulate liquids of several millimetres in size that exceed the length of capillarity, such as a bubble-free liquid capture, and the dropping of that exact amount of liquid. In contrast with previous studies, our model is rigid in the sense that it does not deform due to the surface tension of liquids. This simple and scalable method could be used in laboratories as a pipette for specific purposes, and could be applied as a functional part in soft robotics.

Currently, the manipulatable water size is limited to about 1 cm. This limitation could be overcome by adding elasto-capillarity (i.e. bending deflections of a rib by surface tension) to our structural design, which could lead to an even higher level of performance [21,22]. In future experiments, surface properties of the object should be controlled more carefully by using an appropriate coating technique, which are currently in the planning stages.

## Endnotes

1 In some other species but not species belonging to the genus *Marchantia*, fertilization can also be achieved by transport of sperm mediated by small arthropods [24], or explosive sperm dispersal into the air [25].

2 Although the shaft of the device may contain some liquid [36], it is actually negligible and has little effect on the main results. We confirmed this by repeating the experiments at initial positions with different depths of 9, 20, 25, 30 mm.

## Data accessibility

The experimental movies are available from the journal website in a form of electronic supplementary material. All experimental datasets and CAD data for three-dimensional printer models are available freely on the website: http://www.ritsumei.ac.jp/se/rp/physics/lab/biophys/data.html.
